# Reappearance of Command-Following Is Associated With the Recovery of Language and Internal-Awareness Networks: A Longitudinal Multiple-Case Report

**DOI:** 10.3389/fnsys.2019.00008

**Published:** 2019-02-26

**Authors:** Charlène Aubinet, Rajanikant Panda, Stephen Karl Larroque, Helena Cassol, Mohamed Ali Bahri, Manon Carrière, Sarah Wannez, Steve Majerus, Steven Laureys, Aurore Thibaut

**Affiliations:** ^1^Coma Science Group, Department of GIGA Consciousness and Neurology, University and University Hospital of Liège, Liège, Belgium; ^2^GIGA-Cyclotron Research Centre In Vivo Imaging, University of Liège, Liège, Belgium; ^3^Psychology and Neuroscience of Cognition Research Unit, University of Liège, Liège, Belgium

**Keywords:** minimally conscious state, language, consciousness, positron emission tomography, structural magnetic resonance imaging

## Abstract

The recovery of patients with disorders of consciousness is a real challenge, especially at the chronic stage. After a severe brain injury, patients can regain some slight signs of consciousness, while not being able to functionally communicate. This entity is called the minimally conscious state (MCS), which has been divided into MCS- and MCS+, respectively based on the absence or presence of language-related signs of consciousness. In this series of cases we aimed to describe retrospectively the longitudinal recovery of specific language-related behaviors using neuroimaging measurement in severely brain-injured patients. Among 209 chronic MCS patients admitted to our center from 2008 to 2018, 19 were assessed at two time points by means of behavioral and neuroimaging assessments. Three of them met our inclusion criteria and were diagnosed as MCS- during their first stay and had recovered command-following when they were reassessed (i.e., MCS+). As compared to their first assessments, when the three patients were in a MCS+, they showed less hypometabolism and/or higher gray matter volume in brain regions such as the precuneus and thalamus, as well as the left caudate and temporal/angular cortices known to be involved in various aspects of semantics. According to these preliminary results, the reappearance of language-related behaviors was concomitant with the recovery of metabolism and gray matter in neural regions that have been associated with self-consciousness and language processing. Prospective studies should be conducted to deepen our understanding of the neural correlates of the recovery of language-related behaviors in chronic MCS.

## Introduction

The minimally conscious state (MCS) was defined in 2002 to distinguish unconscious patients from those presenting reproducible behavioral signs of consciousness (Giacino et al., 2002), and was later subcategorized on the basis of the absence or presence of language-related behaviors (Bruno et al., 2011). Patients in MCS- show non-reflexive behaviors, most frequently visual pursuit and fixation, oriented movements or localization to pain (Wannez et al., 2017a). Patients in MCS+ additionally demonstrate command following, intelligible verbalization or intentional communication (Bruno et al., 2011). These criteria require a certain residual language. For example, patients need to be able to understand what is asked in order to respond to verbal commands, and residual semantic processing is necessary.

A left-lateralized network was identified in a review investigating the neural correlates of semantics using functional neuroimaging studies. It is comprised of seven regions: posterior inferior parietal lobule (including angular gyrus), middle temporal gyrus, fusiform and parahippocampal gyri, dorsomedial prefrontal cortex, inferior frontal gyrus, ventromedial prefrontal cortex, and posterior cingulate gyrus (Binder et al., 2009). As regards to severely brain-injured patients, two neuroimaging studies, using either positron emission tomography (PET), or functional magnetic resonance imaging (MRI), demonstrated that MCS+ patients, as compared to MCS- patients, present a higher brain metabolism and functional connectivity in language-related areas, such as Broca’s and Wernicke’s regions or the left fusiform gyrus (Bruno et al., 2012; Aubinet et al., 2018). Nevertheless, it is still undetermined if behavioral improvement from MCS- to MCS+ is solely the consequence of the recovery of language-related functions, and therefore, due to an increase in brain functions in related areas, or if it is the combination of language and consciousness recuperation.

In the present study, we examine whether the MCS sub-categorization also characterizes the trajectory of recovery of individual patients when followed longitudinally. We demonstrate here in three MCS patients the transition from the MCS- to MCS+ based on repeated neuroimaging and behavioral assessments.

## Materials and Methods

Between 2008 and 2018, 209 brain-damaged patients subsequently diagnosed as MCS were admitted into the University Hospital of Liège, including 19 patients who were assessed at two time points using neuroimaging techniques and repeated Coma Recovery Scale-Revised (CRS-R) (Giacino et al., 2004; Wannez et al., 2017b). Inclusion criteria were: >28 days post-injury when first assessed; diagnosis made based on at least 5 CRS-R, diagnosed in MCS- during the first week of assessment and later diagnosed in MCS+ during the second week of assessments. Three patients met our inclusion criteria (age: 23–37 years-old, two TBI, time since onset: 10 months to 5 years). These three patients in MCS- during their first week of assessment, later recovered command following when reassessed during a second week of evaluations (26–31 months later).

The study was approved by the Ethics Committee of the Faculty of Medicine of the University of Liège and written informed consents for study participation and data publication have been obtained from the patients’ legal representatives as well as from the healthy control subjects.

Fluorodeoxyglucose-PET data were acquired on a Gemini TF CT scanner (Philips Medical Systems) and preprocessed as described elsewhere (Thibaut et al., 2012). The data were smoothed with an isotropic 14 mm full-width at half-maximum (FWHM) Gaussian kernel and analyzed using statistical parametric mapping 12 (SPM12). A patient specific template has been used as proposed in a previous study (Phillips et al., 2011). The design matrices included the two repeated scans of each patient and the scans of healthy controls (*n* = 34, age range 19–70 years old, 15 women). Sample *t*-tests were used to assess the fluorodeoxyglucose metabolism differences between patients and controls. Results were considered significant at *p* < 0.05 false discovery rate (FDR)-corrected.

Structural MRI data were obtained with T1-weighted 3D gradient echo sequence (120 slices, repetition time 2.3 s, echo time 2.47 ms, voxel size 1 mm^3^ × 1 mm^3^ × 1.2 mm^3^, flip angle 9°, field of view 256 mm × 256 mm^2^). A T1 voxel-based morphometry (VBM) analysis (Ashburner and Friston, 2000) was carried with VBM8^a^ toolbox, with non-linear modulation and a study-specific DARTEL (Ashburner, 2007) template generated on 50 patients of same population as our patients, other parameters set to defaults. Data were smoothed with a Gaussian kernel of 12 mm FWHM and then modeled using a factorial design with the total intracranial volume as covariate. An exclusive mask of the cerebrospinal fluid mask was used. A design matrix was constructed for each patient, including the two patient’s scans (pre and post) and the scans of MRI-specific control subjects (*n* = 36, age range 20–75 years old, 13 women). Sample *t*-tests were conducted to assess the gray matter differences between patient and controls. Results were considered significant at FDR-corrected *p* < 0.05 voxel-wise over the whole brain.

Statistical analyses identified: (a) brain areas showing hypometabolism (as compared with healthy control subjects) when the patient was in MCS-; (b) brain areas showing hypometabolism when the patient was in MCS+; (c) brain areas showing less hypometabolism or gray matter impairment when the patient was in MCS+ as compared to the scan when the patient was in MCS-.

## Results

**FIGURE 1:**
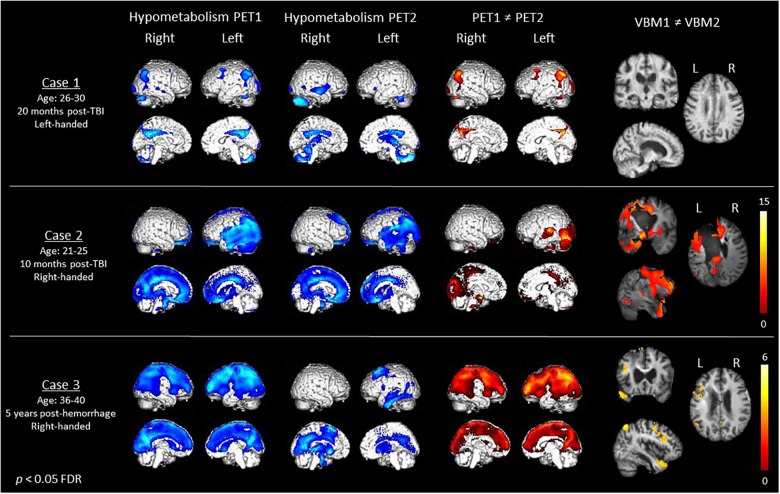
Demographical data (first column), cerebral hypometabolism at Time 1 and Time 2 (second and third column in blue), recovery of metabolism (fourth column in red) as assessed with fluorodeoxyglucose-PET, as well as recovery of gray matter volume (fifth column) as assessed with MRI voxel-based morphometry in the three MCS patients. Color bars represent the T-values.

Demographical and neuroimaging results are presented in Figure 1 and Table 1. Behavioral (i.e., CRS-R sub-scores) and neuroimaging details are reported in Supplementary Materials I, II.

**Table 1:** Coordinates of peak voxels of areas less hypometabolic/atrophic at PET2 as compared to PET1.

	Brain region	*X* (mm)	*Y* (mm)	*Z* (mm)	*p* value^∗^	*Z* value
**Case 1**						
PET1 < PET2	Left precuneus	-4	-56	24	0.001	4.447
	Right angular	44	-66	48	0.006	3.889
	Left angular	-40	-72	44	0.006	3.872
	Left middle occipital gyrus	-34	-92	18	0.009	3.747
	Right inferior occipital gyrus	34	-94	-2	0.020	3.329
	Right cerebellum	38	-72	-26	0.023	3.260
	Left middle frontal gyrus	-32	10	54	0.023	3.249
	Left calcarine gyrus	-6	-102	-2	0.028	3.138
	Right middle frontal gyrus	48	8	52	0.044	2.867
**Case 2**						
PET1 < PET2	Left temporal pole	-32	2	-18	0.000	7.222
	Left cerebellum	-46	-42	-36	0.000	4.847
	Left superior medial frontal gyrus	0	52	20	0.003	3.089
	Right thalamus	16	-14	22	0.006	2.900
VBM1 < VBM2	Left caudate	-8	10	-12	0.000	5.733
	Right caudate	23	9	18	0.001	4.669
	Left temporal fusiform gyrus	-41	-65	-12	0.001	4.115
	Left middle temporal gyrus	-63	-53	-12	0.004	3.566
	Left inferior temporal gyrus	-68	-27	-20	0.006	3.430
	Left angular gyrus	-50	-65	39	0.029	2.672
**Case 3**						
PET1 < PET2	Left middle frontal gyrus	-40	4	56	0.000	6.147
	Right caudate	18	-8	24	0.001	3.505
	Left caudate	-14	8	14	0.002	3.346
VBM1 < VBM2	Right thalamus	17	-7	14	0.019	3.961
	Left inferior temporal gyrus	-66	-56	-9	0.019	3.928
	Left precuneus	2	-42	59	0.019	3.901
	Left superior frontal gyrus	-11	17	63	0.019	3.792
	Right middle temporal gyrus	66	-51	-2	0.020	3.759
	Right parahippocampal gyrus	21	-4	-18	0.020	3.745
	Left superior temporal gyrus	-51	18	-30	0.020	3.718
	Left parahippocampal gyrus	-23	-24	-35	0.020	3.638
	Left precentral gyrus	-38	-4	62	0.021	3.509
	Left inferior parietal lobule	-44	-68	51	0.021	3.495
	Left parahippocampal gyrus	-24	-24	-12	0.023	3.335
	Left caudate	-17	9	15	0.024	3.240
	Right middle frontal gyrus	32	48	3	0.027	3.123


^*^FDR corrected. ^a^http://www.neuro.uni-jena.de/vbm/.

At Time 1, the left-handed case 1 presented hypometabolism in the bilateral angular, fusiform and middle frontal gyri, left calcarine gyrus and right thalamus, cerebellum, inferior occipital, and middle temporal gyri. When this patient was able to respond to simple commands 27 months later, we identified a significant recovery of metabolism in the bilateral angular and middle frontal gyri, left precuneus, middle occipital and calcarine gyri, right inferior occipital gyrus and cerebellum. Gray matter volume did not significantly increase or decrease between both examinations.

At Time 1, Case 2 presented hypometabolism mostly in the left superior medial gyrus and temporo-parieto-occipital cortex. Thirty-one months later, we observed command following, along with a significant increase of metabolism in the left temporal lobule, cerebellum and superior medial gyrus, as well as the thalamus. Gray matter volume was shown to be significantly increased in MCS+ than in MCS- in the bilateral caudate and the left fusiform, angular and middle/inferior temporal gyri.

Finally, at Time 1, case 3 presented hypometabolism in the left middle frontal and temporal gyri, left inferior parietal lobule, left rolandic operculum, and right thalamus. When he had recovered command following 26 months later, we identified a recovery of metabolism in the bilateral caudate and the left middle frontal gyrus. Between the two scans, gray matter volume was found to be more preserved in bilateral parahippocampal gyri, in the left inferior and superior temporal cortex, caudate, inferior parietal lobule, precuneus and frontal cortex, as well as in the right thalamus, middle frontal, and temporal cortex.

## Discussion

The present findings offer a new perspective to understand the neural correlates of the recovery of language-related behaviors, and more specifically command following (behavior recovered in all three patients). We here report that the recovery of this ability in three chronic MCS patients is paralleled with an increase in brain metabolism and gray matter in regions previously related to language and/or consciousness.

First, all patients presented an increase of brain metabolism in regions that have been associated with language processing. For example, Binder et al. (2009) considered the left angular gyrus as particularly involved in semantics (i.e., sentence comprehension, complex information integration and knowledge retrieval), and this region was less hypometabolic when case 1 was MCS+ as compared to MCS-. Moreover, a recovery of its contralateral homolog was reported. According to the same authors, the left temporal areas highlighted in case 2 were also shown to be activated in language tasks and to support concept retrieval. Finally, case 3 presented a better metabolism in the left caudate, which was associated with language control skills (Crinion et al., 2006; Friederici, 2006). Using structural MRI, patients 2 and 3 also showed a larger gray matter volume in language comprehension areas (i.e., left fusiform, angular, and temporal cortex) (Binder et al., 2009) when having recovered command following. Thus our findings are congruent with previous studies in MCS patients, showing a relationship between the ability to respond to commands and left hemisphere functions (Bruno et al., 2012; Aubinet et al., 2018). Nevertheless, we found that the structural and functional changes between MCS- and MCS+ might be bilateral. The non-dominant hemisphere could therefore contribute to the recovery of command following. This is also consistent with previous studies showing compensatory mechanisms in the contralateral right hemisphere in conscious aphasic patients (Teki et al., 2013; Artzi et al., 2016).

Furthermore, the thalamus and/or precuneus showed an improvement of metabolism in the two first patients and an increase of gray matter structure in the third case. These regions are critical for consciousness processes, and especially self-consciousness as they are part of the internal consciousness network (Vanhaudenhuyse et al., 2011; Di Perri et al., 2016). Consequently, our results tend to confirm that, besides language processing differences, MCS- and MCS+ categories also reflect differentiated levels of consciousness.

It is however important to note that other higher cognitive functions might be involved in the sub-categorization of the MCS. Indeed, for all three cases an improvement was shown in various frontal areas related to executive functions (Miyake et al., 2000), or in regions such as the caudate, which is implicated in learning or cognitive control (Chiu et al., 2017). These skills could also be involved in the recovery of command following as assessed with the CRS-R.

Our results are not representative of the population and prospective studies including a large sample of patients are needed in order to overcome statistical limitations. Future longitudinal studies should also investigate the clinical implications on long-term outcomes of the transition from MCS- to MCS+, as it could be hypothesized that patients in MCS+ could have a better outcome than patients in MCS-, making the distinction between these two entities crucial. In addition, single subject VBM analysis are prone to false positive/type 1 error and group studies may limit this risk of false positive. Nevertheless, our findings point-out the possibility of command following recovery even in chronic MCS- patients, which seems in line with a recovery of brain functions in the ipsi- or contra-lateral language networks, as well as in the consciousness network. This is particularly relevant given the importance of this behavior for a potential communication (Giacino et al., 2002, 2004).

## Conclusion

The reappearance of command following in the three patients was concomitant with a recovery of metabolism and gray matter structure in language-related areas, mainly in the left temporal lobule, angular gyrus, fusiform gyrus, and caudate. It was also concomitant with functional and structural recovery of structures such as the thalamus and the precuneus involved in self-consciousness. The present results indicate that the transition from MCS- to MCS+ involves recovery in networks particularly associated with both language and consciousness.

## Author Contributions

CA, SW, and AT conceived and planned the presented research. CA, HC, and MC contributed to data acquirement. CA, RP, SL, MB, SM, SL, and AT worked on data analyses and interpretation. CA drafted the manuscript under AT’s supervision. All authors provided critical feedback and helped to shape the manuscript.

## Conflict of Interest Statement

The authors declare that the research was conducted in the absence of any commercial or financial relationships that could be construed as a potential conflict of interest.
