# Supplementary material for: Reappearance of Command-Following Is Associated With the Recovery of Language and Internal-Awareness Networks: A Longitudinal Multiple-Case Report
Source: Front Syst Neurosci. 2019 Feb 26;13:8. doi: 10.3389/fnsys.2019.00008 (PMC6399132; doi:10.3389/fnsys.2019.00008)
Supplement: Supplementary file 1 [file Table_1.DOCX]

*Reappearance of command-following is associated to recovery of language and consciousness networks: A longitudinal multiple-case report*

Charlène Aubinet¹*, Rajanikant Panda¹, Stephen Karl Larroque¹, Helena Cassol¹, Mohamed Ali Bahri**²**, Manon Carrière¹, Sarah Wannez¹, Steve Majerus**³**, Steven Laureys¹, Aurore Thibaut¹

Supplementary material

I – Behavioral assessments based on repeated Coma Recovery Scale-Revised.

II – Clusters emerging from the neuroimaging analyses

**Supplementary material I. Behavioral assessments based on repeated Coma Recovery Scale-Revised.**

|  | **Case 1** | | **Case 2** | | **Case 3** | |
| --- | --- | --- | --- | --- | --- | --- |
|  | **1^st^** | **2^nd^** | **1^st^** | **2^nd^** | **1^st^** | **2^nd^** |
| **AUDITORY FUNCTION** |  |  |  |  |  |  |
| 4 – Consistent Movement to Command* |  |  |  |  |  |  |
| 3 – Reproducible Movement to Command* |  | X |  | X |  | X |
| 2 – Localization to Sound | X |  |  |  |  | X |
| 1 – Auditory Startle | X |  | X | X | X | X |
| 0 – None |  |  |  |  |  |  |
| **VISUAL FUNCTION SCALE** |  |  |  |  |  |  |
| 5 – Object Recognition* |  |  |  |  |  |  |
| 4 – Object Localization: Reaching* |  |  |  |  |  |  |
| 3 – Pursuit Eye Movements* |  | X | X | X | X | X |
| 2 – Fixation* | X |  |  |  |  |  |
| 1 – Visual Startle |  |  | X | X | X | X |
| 0 – None |  |  |  |  |  |  |
| **MOTOR FUNCTION SCALE** |  |  |  |  |  |  |
| 6 – Functional Object Use ^†^ |  |  |  |  |  |  |
| 5 – Automatic Motor Response * |  |  |  |  |  | X |
| 4 – Object Manipulation* |  |  |  |  |  |  |
| 3 – Localization to Noxious Stimulation* |  |  |  |  | X |  |
| 2 – Flexion Withdrawal | X | X | X | X | X | X |
| 1 – Abnormal Posturing | X | X |  |  |  | X |
| 0 – None/Flaccid |  |  |  |  |  |  |
| **OROMOTOR/ VERBAL FUNCTION SCALE** |  |  |  |  |  |  |
| 3 – Intelligible Verbalization* |  |  |  |  |  |  |
| 2 – Vocalization/Oral Movement | X | X |  | X | X | X |
| 1 – Oral Reflexive Movement | X | X | X | X |  |  |
| 0 – None |  |  |  |  |  |  |
| **COMMUNICATION SCALE** |  |  |  |  |  |  |
| 2 – Functional: Accurate ^†^ |  |  |  |  |  |  |
| 1 – Non-Functional: intentional* |  |  |  |  |  |  |
| 0 – None | X | X | X | X | X | X |
| **AROUSAL SCALE** |  |  |  |  |  |  |
| 3 – Attention |  |  |  |  |  |  |
| 2 – Eye Opening w/o Stimulation |  | X | X | X | X | X |
| 1 – Eye Opening with Stimulation | X | X | X | X | X | X |
| 0 – Unarousable |  |  |  |  |  |  |
| DIAGNOSIS | MCS- | MCS+ | MCS- | MCS+ | MCS- | MCS+ |

MCS = minimally conscious state; * = behavioral sign of MCS; ^†^ = behavioral sign of emergence from the MCS.

**Supplementary material II. Clusters emerging from the neuroimaging analyses.**

|  | **Cluster** | | | **Peak** | | | | **Coordinates** | | |
| --- | --- | --- | --- | --- | --- | --- | --- | --- | --- | --- |
|  | *p*(FWE-corr) | equiv *k* | *p*(unc) | *p*(FWE-corr) | *p*(FDR-corr) | T | equiv *Z* | *x* | *y* | *z* |
| **Case 1** | | | | | | | | | | |
| PET1 | 0.026 | 2666 | 0.008 | 0.000 | 0.000 | 7.349 | 5.762 | 4 | -48 | 28 |
| hypometabolism | 0.092 | 1659 | 0.031 | 0.024 | 0.001 | 5.026 | 4.373 | 10 | -72 | -50 |
|  | 0.651 | 291 | 0.336 | 0.090 | 0.004 | 4.510 | 4.011 | -4 | -104 | 0 |
|  | 0.804 | 135 | 0.520 | 0.124 | 0.006 | 4.373 | 3.911 | 32 | -98 | -2 |
|  | 0.218 | 1037 | 0.078 | 0.133 | 0.006 | 4.344 | 3.889 | 44 | -66 | 48 |
|  | 0.137 | 1367 | 0.047 | 0.140 | 0.006 | 4.320 | 3.872 | -40 | -72 | 44 |
|  | 0.269 | 893 | 0.100 | 0.186 | 0.008 | 4.195 | 3.778 | -28 | -46 | -8 |
|  | 0.903 | 41 | 0.744 | 0.564 | 0.021 | 3.594 | 3.313 | 14 | -24 | 10 |
|  | 0.571 | 383 | 0.270 | 0.628 | 0.023 | 3.513 | 3.249 | -32 | 10 | 54 |
|  | 0.929 | 18 | 0.843 | 0.653 | 0.024 | 3.482 | 3.223 | -10 | 16 | 30 |
|  | 0.896 | 47 | 0.724 | 0.820 | 0.033 | 3.250 | 3.034 | 70 | -26 | 0 |
|  | 0.924 | 22 | 0.823 | 0.854 | 0.036 | 3.194 | 2.987 | 32 | -44 | -8 |
|  | 0.905 | 39 | 0.751 | 0.898 | 0.041 | 3.106 | 2.913 | 66 | -8 | -12 |
|  | 0.928 | 19 | 0.838 | 0.921 | 0.044 | 3.051 | 2.867 | 48 | 8 | 52 |
| PET2 | 0.109 | 2022 | 0.028 | 0.000 | 0.000 | 7.176 | 5.670 | 4 | -34 | 32 |
| hypometabolism | 0.196 | 1507 | 0.053 | 0.000 | 0.000 | 6.721 | 5.423 | 34 | -42 | -6 |
|  | 0.001 | 7461 | 0.000 | 0.000 | 0.000 | 6.518 | 5.307 | -28 | -46 | -6 |
|  | 0.257 | 1276 | 0.073 | 0.031 | 0.001 | 4.933 | 4.309 | 66 | -2 | -4 |
|  | 0.937 | 74 | 0.675 | 0.231 | 0.004 | 4.094 | 3.703 | -4 | -104 | 0 |
|  | 0.942 | 65 | 0.698 | 0.324 | 0.005 | 3.925 | 3.573 | 32 | -98 | -2 |
|  | 0.966 | 24 | 0.831 | 0.344 | 0.006 | 3.894 | 3.549 | 38 | -14 | -8 |
|  | 0.935 | 76 | 0.670 | 0.498 | 0.009 | 3.678 | 3.380 | -14 | -18 | 20 |
|  | 0.958 | 39 | 0.774 | 0.643 | 0.013 | 3.495 | 3.234 | 34 | 62 | -12 |
|  | 0.939 | 70 | 0.685 | 0.697 | 0.014 | 3.425 | 3.177 | -54 | 18 | 0 |
|  | 0.960 | 35 | 0.788 | 0.842 | 0.022 | 3.213 | 3.003 | 64 | -58 | 2 |
|  | 0.974 | 11 | 0.896 | 0.925 | 0.031 | 3.040 | 2.858 | 30 | 28 | -24 |
|  | 0.978 | 6 | 0.929 | 0.933 | 0.032 | 3.018 | 2.839 | -28 | -100 | -4 |
|  | 0.975 | 9 | 0.908 | 0.944 | 0.035 | 2.983 | 2.810 | -30 | 22 | -34 |
|  | 0.980 | 3 | 0.955 | 0.967 | 0.041 | 2.891 | 2.731 | 0 | -18 | 20 |
|  | 0.982 | 1 | 0.978 | 0.980 | 0.048 | 2.811 | 2.663 | -32 | 62 | -8 |
|  | 0.982 | 1 | 0.978 | 0.981 | 0.048 | 2.809 | 2.661 | 58 | -8 | -38 |
|  | 0.982 | 1 | 0.978 | 0.983 | 0.050 | 2.792 | 2.646 | 60 | -4 | -34 |
| PET1 < PET2 | 0.183 | 1160 | 0.064 | 0.018 | 0.001 | 5.135 | 4.447 | -4 | -56 | 24 |
|  | 0.225 | 1015 | 0.081 | 0.133 | 0.006 | 4.344 | 3.889 | 44 | -66 | 48 |
|  | 0.137 | 1367 | 0.047 | 0.140 | 0.006 | 4.320 | 3.872 | -40 | -72 | 44 |
|  | 0.751 | 187 | 0.444 | 0.204 | 0.009 | 4.152 | 3.747 | -34 | -92 | 18 |
|  | 0.766 | 172 | 0.464 | 0.421 | 0.016 | 3.780 | 3.461 | -34 | -56 | -22 |
|  | 0.872 | 70 | 0.656 | 0.548 | 0.020 | 3.614 | 3.329 | 34 | -94 | -2 |
|  | 0.772 | 166 | 0.472 | 0.617 | 0.023 | 3.528 | 3.260 | 38 | -72 | -26 |
|  | 0.571 | 383 | 0.270 | 0.628 | 0.023 | 3.513 | 3.249 | -32 | 10 | 54 |
|  | 0.936 | 12 | 0.878 | 0.733 | 0.028 | 3.378 | 3.138 | -6 | -102 | -2 |
|  | 0.947 | 4 | 0.939 | 0.879 | 0.039 | 3.146 | 2.947 | 70 | -28 | 2 |
|  | 0.949 | 3 | 0.950 | 0.890 | 0.040 | 3.123 | 2.927 | -10 | -104 | -6 |
|  | 0.944 | 6 | 0.921 | 0.902 | 0.041 | 3.097 | 2.906 | -2 | -100 | -2 |
|  | 0.951 | 2 | 0.961 | 0.905 | 0.041 | 3.091 | 2.901 | -6 | -104 | 10 |
|  | 0.949 | 3 | 0.950 | 0.909 | 0.042 | 3.081 | 2.893 | -22 | -58 | -12 |
|  | 0.928 | 19 | 0.838 | 0.921 | 0.044 | 3.051 | 2.867 | 48 | 8 | 52 |
|  | 0.951 | 2 | 0.961 | 0.926 | 0.044 | 3.038 | 2.856 | 16 | -84 | -32 |
|  | 0.953 | 1 | 0.975 | 0.940 | 0.047 | 2.996 | 2.821 | -10 | -104 | 4 |
|  | 0.953 | 1 | 0.975 | 0.953 | 0.049 | 2.951 | 2.783 | 12 | -82 | -32 |
| **Case 2** | | | | | | | | | | |
| PET1 | 0.000 | 43552 | 0.000 | 0.000 | 0.000 | 17.596 | 65535 | -10 | 50 | 6 |
| hypometabolism | 1.000 | 10 | 0.943 | 0.000 | 0.000 | 7.687 | 5.934 | -24 | 18 | -36 |
|  | 0.997 | 177 | 0.673 | 0.019 | 0.000 | 5.035 | 4.380 | -8 | -28 | -46 |
|  | 0.998 | 138 | 0.715 | 0.765 | 0.004 | 3.234 | 3.020 | 10 | -76 | -50 |
|  | 1.000 | 7 | 0.955 | 0.921 | 0.008 | 2.949 | 2.781 | 36 | 62 | -10 |
|  | 1.000 | 16 | 0.923 | 0.986 | 0.017 | 2.654 | 2.527 | 44 | -44 | -50 |
|  | 1.000 | 3 | 0.974 | 0.992 | 0.020 | 2.588 | 2.469 | 18 | -102 | -12 |
|  | 1.000 | 2 | 0.980 | 0.998 | 0.028 | 2.431 | 2.330 | 34 | 64 | -4 |
|  | 1.000 | 1 | 0.988 | 1.000 | 0.045 | 2.229 | 2.150 | -2 | -34 | 4 |
|  | 1.000 | 1 | 0.988 | 1.000 | 0.046 | 2.212 | 2.135 | 20 | -100 | -14 |
|  | 1.000 | 1 | 0.988 | 1.000 | 0.049 | 2.187 | 2.112 | 32 | 64 | -8 |
|  | 1.000 | 1 | 0.988 | 1.000 | 0.049 | 2.185 | 2.111 | 42 | 54 | -14 |
| PET2 | 0.000 | 36836 | 0.000 | 0.000 | 0.000 | 17.321 | 65535 | -10 | 50 | 4 |
| hypometabolism | 0.999 | 10 | 0.939 | 0.000 | 0.000 | 7.723 | 5.952 | -24 | 18 | -36 |
|  | 0.994 | 172 | 0.658 | 0.000 | 0.000 | 6.461 | 5.274 | 46 | -44 | -46 |
|  | 1.000 | 4 | 0.966 | 0.969 | 0.015 | 2.777 | 2.633 | 18 | -102 | -12 |
|  | 0.999 | 8 | 0.947 | 0.996 | 0.029 | 2.500 | 2.391 | 16 | -40 | 78 |
|  | 1.000 | 1 | 0.987 | 1.000 | 0.049 | 2.266 | 2.183 | 18 | -14 | 28 |
| PET1 < PET2 | 0.040 | 8825 | 0.005 | 0.000 | 0.000 | 10.695 | 7.222 | -32 | 2 | -18 |
|  | 0.999 | 45 | 0.853 | 0.000 | 0.000 | 9.206 | 6.636 | -46 | -18 | -38 |
|  | 0.997 | 193 | 0.657 | 0.003 | 0.000 | 5.751 | 4.847 | -46 | -42 | -36 |
|  | 0.997 | 177 | 0.673 | 0.019 | 0.000 | 5.035 | 4.380 | -8 | -28 | -46 |
|  | 0.997 | 182 | 0.668 | 0.021 | 0.000 | 4.991 | 4.349 | 6 | 50 | -26 |
|  | 0.998 | 117 | 0.740 | 0.130 | 0.000 | 4.259 | 3.826 | 16 | 12 | -22 |
|  | 1.000 | 1 | 0.988 | 0.280 | 0.001 | 3.906 | 3.559 | -6 | 2 | -20 |
|  | 0.999 | 85 | 0.784 | 0.291 | 0.001 | 3.885 | 3.542 | 0 | -28 | 0 |
|  | 1.000 | 33 | 0.879 | 0.705 | 0.003 | 3.317 | 3.089 | 0 | 52 | 20 |
|  | 0.998 | 138 | 0.715 | 0.765 | 0.004 | 3.234 | 3.020 | 10 | -76 | -50 |
|  | 1.000 | 25 | 0.898 | 0.783 | 0.004 | 3.208 | 2.999 | -4 | 0 | -18 |
|  | 1.000 | 19 | 0.914 | 0.855 | 0.006 | 3.090 | 2.900 | 16 | -14 | 22 |
|  | 1.000 | 7 | 0.955 | 0.921 | 0.008 | 2.949 | 2.781 | 36 | 62 | -10 |
|  | 1.000 | 5 | 0.964 | 0.953 | 0.011 | 2.847 | 2.694 | 18 | 66 | -6 |
|  | 1.000 | 1 | 0.988 | 0.996 | 0.025 | 2.491 | 2.383 | 4 | 60 | 16 |
|  | 1.000 | 1 | 0.988 | 0.996 | 0.025 | 2.486 | 2.379 | 14 | -2 | -10 |
|  | 1.000 | 1 | 0.988 | 0.997 | 0.026 | 2.473 | 2.367 | 8 | -6 | 52 |
|  | 1.000 | 1 | 0.988 | 0.997 | 0.028 | 2.445 | 2.343 | -4 | -12 | -14 |
|  | 1.000 | 2 | 0.980 | 0.998 | 0.028 | 2.431 | 2.330 | 34 | 64 | -4 |
|  | 1.000 | 1 | 0.988 | 0.998 | 0.029 | 2.430 | 2.330 | -16 | -14 | -30 |
|  | 1.000 | 1 | 0.988 | 0.998 | 0.029 | 2.416 | 2.317 | -24 | -60 | -22 |
|  | 1.000 | 1 | 0.988 | 0.998 | 0.031 | 2.394 | 2.298 | -18 | -38 | -20 |
|  | 1.000 | 1 | 0.988 | 0.999 | 0.032 | 2.385 | 2.290 | 12 | -6 | -12 |
|  | 1.000 | 1 | 0.988 | 0.999 | 0.039 | 2.295 | 2.210 | 28 | 66 | -4 |
|  | 1.000 | 1 | 0.988 | 1.000 | 0.045 | 2.230 | 2.151 | 2 | 54 | 28 |
|  | 1.000 | 1 | 0.988 | 1.000 | 0.045 | 2.230 | 2.151 | -2 | -60 | 40 |
|  | 1.000 | 1 | 0.988 | 1.000 | 0.045 | 2.229 | 2.150 | -2 | -34 | 4 |
|  | 1.000 | 1 | 0.988 | 1.000 | 0.046 | 2.212 | 2.135 | 20 | -100 | -14 |
|  | 1.000 | 1 | 0.988 | 1.000 | 0.048 | 2.196 | 2.120 | 22 | 28 | -24 |
|  | 1.000 | 1 | 0.988 | 1.000 | 0.049 | 2.187 | 2.112 | 32 | 64 | -8 |
|  | 1.000 | 1 | 0.988 | 1.000 | 0.049 | 2.185 | 2.111 | 42 | 54 | -14 |
| VBM1< VBM2 | 0.000 | 44386 | 0.000 | 0.001 | 0.000 | 15.207 | 5.733 | -8 | 10 | -12 |
|  | 1.000 | 103 | 0.646 | 0.050 | 0.001 | 8.669 | 4.669 | 23 | 9 | 18 |
|  | 0.960 | 696 | 0.210 | 0.273 | 0.001 | 6.597 | 4.115 | -41 | -65 | -12 |
|  | 0.999 | 277 | 0.430 | 0.775 | 0.004 | 5.066 | 3.566 | -63 | -53 | -12 |
|  | 0.999 | 236 | 0.468 | 0.878 | 0.006 | 4.745 | 3.430 | -68 | -27 | -20 |
|  | 1.000 | 173 | 0.540 | 1.000 | 0.029 | 3.263 | 2.672 | -50 | -65 | 39 |
| **Case 3** | | | | | | | | | | |
| PET1 | 0.000 | 71242 | 0.000 | 0.000 | 0.000 | 8.122 | 6.147 | -40 | 4 | 56 |
| hypometabolism | 1.000 | 4 | 0.971 | 1.000 | 0.039 | 2.197 | 2.121 | 14 | -24 | 18 |
|  | 1.000 | 2 | 0.982 | 1.000 | 0.042 | 2.154 | 2.082 | 10 | -18 | 20 |
|  | 1.000 | 1 | 0.989 | 1.000 | 0.049 | 2.068 | 2.004 | -58 | -8 | 18 |
| PET2 | 0.000 | 18568 | 0.000 | 0.000 | 0.000 | 10.561 | 7.173 | -12 | -22 | 8 |
| hypometabolism | 0.985 | 38 | 0.800 | 0.001 | 0.000 | 6.144 | 5.088 | -18 | -26 | 18 |
|  | 0.993 | 5 | 0.944 | 0.103 | 0.001 | 4.450 | 3.967 | -12 | -20 | -12 |
|  | 0.994 | 1 | 0.980 | 0.540 | 0.006 | 3.622 | 3.335 | -8 | -20 | 20 |
|  | 0.974 | 80 | 0.693 | 0.728 | 0.010 | 3.382 | 3.142 | -62 | 6 | 12 |
|  | 0.981 | 53 | 0.757 | 0.867 | 0.017 | 3.167 | 2.965 | -56 | -6 | 48 |
| PET1 < PET2 | 0.000 | 59167 | 0.000 | 0.000 | 0.000 | 8.122 | 6.147 | -40 | 4 | 56 |
|  | 1.000 | 48 | 0.858 | 0.380 | 0.001 | 3.837 | 3.505 | 18 | -8 | 24 |
|  | 1.000 | 105 | 0.773 | 0.530 | 0.002 | 3.635 | 3.346 | -14 | 8 | 14 |
|  | 1.000 | 3 | 0.976 | 0.999 | 0.023 | 2.450 | 2.347 | -20 | 12 | 2 |
|  | 1.000 | 4 | 0.971 | 1.000 | 0.039 | 2.197 | 2.121 | 14 | -24 | 18 |
|  | 1.000 | 1 | 0.989 | 1.000 | 0.042 | 2.157 | 2.085 | -12 | -36 | 46 |
|  | 1.000 | 2 | 0.982 | 1.000 | 0.042 | 2.154 | 2.082 | 10 | -18 | 20 |
|  | 1.000 | 1 | 0.989 | 1.000 | 0.047 | 2.090 | 2.025 | -26 | -64 | -16 |
|  | 1.000 | 1 | 0.989 | 1.000 | 0.049 | 2.068 | 2.004 | -58 | -8 | 18 |
| VBM1 < VBM2 | 0.998 | 216 | 0.418 | 0.431 | 0.019 | 6.126 | 3.961 | 17 | -7 | 14 |
|  | 0.082 | 3092 | 0.006 | 0.463 | 0.019 | 6.030 | 3.928 | -66 | -56 | -9 |
|  | 0.049 | 3580 | 0.003 | 0.490 | 0.019 | 5.952 | 3.901 | 2 | -42 | 59 |
|  | 0.980 | 402 | 0.267 | 0.602 | 0.019 | 5.647 | 3.792 | -11 | 17 | 63 |
|  | 0.963 | 480 | 0.226 | 0.636 | 0.020 | 5.558 | 3.759 | 66 | -51 | -2 |
|  | 0.436 | 1546 | 0.039 | 0.650 | 0.020 | 5.522 | 3.745 | 21 | -4 | -18 |
|  | 0.608 | 1207 | 0.064 | 0.677 | 0.020 | 5.451 | 3.718 | -51 | 18 | -30 |
|  | 0.992 | 304 | 0.334 | 0.755 | 0.020 | 5.246 | 3.638 | -23 | -24 | -35 |
|  | 0.276 | 1974 | 0.022 | 0.862 | 0.021 | 4.931 | 3.509 | -38 | -4 | 62 |
|  | 0.588 | 1243 | 0.061 | 0.872 | 0.021 | 4.896 | 3.495 | -44 | -68 | 51 |
|  | 1.000 | 128 | 0.540 | 0.954 | 0.023 | 4.533 | 3.335 | -24 | -24 | -12 |
|  | 1.000 | 129 | 0.539 | 0.979 | 0.024 | 4.329 | 3.240 | -17 | 9 | 15 |
|  | 0.998 | 223 | 0.410 | 0.994 | 0.027 | 4.090 | 3.123 | 32 | 48 | 3 |
